# A Practical Approach for Environmental Flow Calculation to Support Ecosystem Management in Wujiang River, China

**DOI:** 10.3390/ijerph191811615

**Published:** 2022-09-15

**Authors:** Xiaokuan Ni, Zengchuan Dong, Wei Xie, Shujun Wu, Mufeng Chen, Hongyi Yao, Wenhao Jia

**Affiliations:** 1 College of Hydrology and Water Resources, Hohai University, Nanjing 210024, China; 2 Department of Water Resources and Ecosystems, UNESCO-IHE Institute for Water Education, 2611AX Delft, The Netherlands; 3 Department of Hydropower and Water Conservancy Engineering, POWERCHINA Huadong Engineering Corporation Limited, Hangzhou 311122, China; 4 Department of Aquatic Ecosystem Analysis and Management, Helmholtz Centre for Environmental Research—UFZ, 39104 Magdeburg, Germany; 5 Department of Civil Engineering, The University of Hong Kong, Hong Kong, China; 6 Pearl River Water Resources Research Institute, Guangzhou 510611, China

**Keywords:** environmental flow, hierarchical target, tennant method, flow duration curve, Wujiang River Basin

## Abstract

To promote ecosystem protection in the Wujiang River, this paper proposes a practical approach for calculating the environmental flow. The proposed approach combines the idea of the “guarantee rate” of the flow duration curve (FDC) method and the grading idea of the Tennant method. A daily flow series of the Wujiang River was compiled from 1956 to 2019 and used to compare the effect of the proposed approach versus the traditional approaches in four selected sections along the river. The results show that the environmental flow of the Wujiang River can be divided into five levels by the T-FDC method, with a level-by-level disparity, and all levels can capture the temporal and spatial variability of river flow. Additionally, the calculated basic environmental flow process ranges between the historical minimum and second minimum monthly average flow, and the threshold width of the optimal flow is more reasonable than the Tennant method. The T-FDC method can provide technical support for Wujiang River ecosystem management and sustainable development.

## 1. Introduction

The impact of reservoir development on river ecosystems is of great concern in practice. River hydrological processes have essential ecological effects on river channels and their surrounding organisms [1,2]. While reservoirs regulate runoff for flood control, power generation, and irrigation, they also change the ecological situation of the river to an extent, causing some stress to the ecosystem [3,4]. How to mitigate or eliminate the negative impacts of cascade reservoirs on river ecology is a complex systemic issue [5].

With the global emphasis on ecological protection, hydraulic projects are increasingly required to address and account for river ecological functions in project design and operation. Studies and practices show that one of the essential measures to reduce the ecological impact of hydraulic projects, such as dams and reservoirs in rivers, is to take into account the ecological needs of the river by optimizing the operation of those projects, or the so-called “ecological operation” [6,7,8,9]. To formulate ecological operation schemes, a prerequisite is identifying and characterizing the ecological requirement on the amount of river flow, known as environmental flow, so that it can be incorporated in or accounted for in the operation of hydraulic projects and maintain downstream health.

However, the cascade development of the Wujiang River Basin in southwest China started long ago, mainly to generate electricity, and thus limited consideration of river ecology. Most reservoir projects have no targets for maintaining environmental flow when beginning the design [10]. An easy-to-use and reasonable method for environmental flow calculation is urgently needed to manage ecological operations. Determining rivers’ environmental flow requirement is also a research hotspot in ecology, hydrology, and water resources [11,12,13].

Depending on their focus, available methods for calculating the environmental flow of rivers can be divided into four main categories: (1) hydrological methods using historical hydrological data, (2) hydraulic methods based on the hydraulic properties of river sections, (3) habitat simulation methods built on habitat suitability analysis, and (4) holistic methods comprehensively considering various factors [14,15]. The hydrological methods are most widely used globally due to their many advantages, such as simple and easy operation, only needing flow data, avoidance of expensive field observations, strong versatility, and quick determination of value. According to some estimates, there are more than 200 calculation methods for environmental flow, and hydrological methods account for about 30% [16,17].

The hydrological methods include the Tennant method [18], the Tessman method [19], the indicators of hydrologic alteration/range of variability approach (IHA/RVA) method [20], the flow duration curve (FDC) method [21] and so on, among which the two most commonly used are the Tennant method and the FDC method. The Tennant method, based on the habits of aquatic organisms, divides a natural year into a fish spawning period and a general water use period [22]. It relates river flow to fish habitat quality, links varying ecological status to different flow levels of rivers, and then sets up the targets of ecological river flow. The FDC is a statistical characteristic curve drawn based on the duration or frequency of the flow equal to or exceeding a particular value during the observation time. As the proportion of time when a specific flow exceeds all historical records, the FDC more adequately reflects the runoff characteristics of the basin under various flow states from low to high [23].

A common problem found for hydrological methods is that they ignore the temporal and spatial variation in river flow and do not capture or reflect the hydrological variation in natural river flow. In addition, the basis of ecological status grading is a fixed percentage, which lacks physical connotation. The methods’ poor spatial transferability and vulnerability to extreme flow events are also issues that need to be solved [16,24,25]. Given these shortcomings of hydrological methods, many scholars proposed improvement strategies from three aspects: characteristic flow, calculation period, and percentage coefficient. Examples include using the annual flow of a typical year or monthly average flow instead of multi-year average flow [26], revising the division of periods based on the seasonal characteristics of the river ecosystem and the activity cycle of the biological population [27], calibrating the relationship between river flow and ecological health based on actual local conditions [28], and so on.

Although factors affecting environmental flow are considered more and more carefully, those improved hydrological methods [29] also make the calculation more complex and lose the essential simplicity of hydrological methods. Furthermore, it is difficult to predict the water regime at the beginning of the year in the actual operation and management of river ecological protection [30]. This makes the improved methods for calculating environmental flow challenging to operate in a practical way that considers the interannual variation in water regimes and distinguishes the wet, normal, and dry years. The technique adopted should not be too complicated from the perspective of practice.

In this paper, we propose and explore an alternative method for calculating the environmental flow of the Wujiang River, called the “Tennant-FDC Method” (T-FDC), to provide technical support for its healthy development. The development of the T-FDC method explicitly considers the annual variation in the river hydrological process combined with the advantage of the FDC method to truly reflect the runoff characteristics under each flow state and the classification idea of the Tennant method. As such, the T-FDC method captures the monthly variability of river flow and is spatially transferable.

The rest of this paper is organized as follows: Section 2 describes the regional situation and requirements of the Wujiang River Basin in southwest China. Section 3 describes the steps of the T-FDC method to calculate multi-level environmental flow. Section 4 shows the results and compares them with other hydrological methods. Section 5 concludes the paper with some discussion.

## 2. Study Area and Datasets

The Wujiang River Basin is located at 104°18′~109°22′ E and 26°07′~30°22′ N, involving four provinces of Yunnan, Guizhou, Chongqing, and Hubei in China, with a total area of 87,920 km^2^ and mainstream length of 1037 km. It is the largest tributary on the south bank of the upper Yangtze River and a representative river in southwest China. Its runoff is mainly influenced by rainfall, with a clear distinction between wet and dry, with May to September accounting for about 80% of the annual [31].

**Figure 1:**
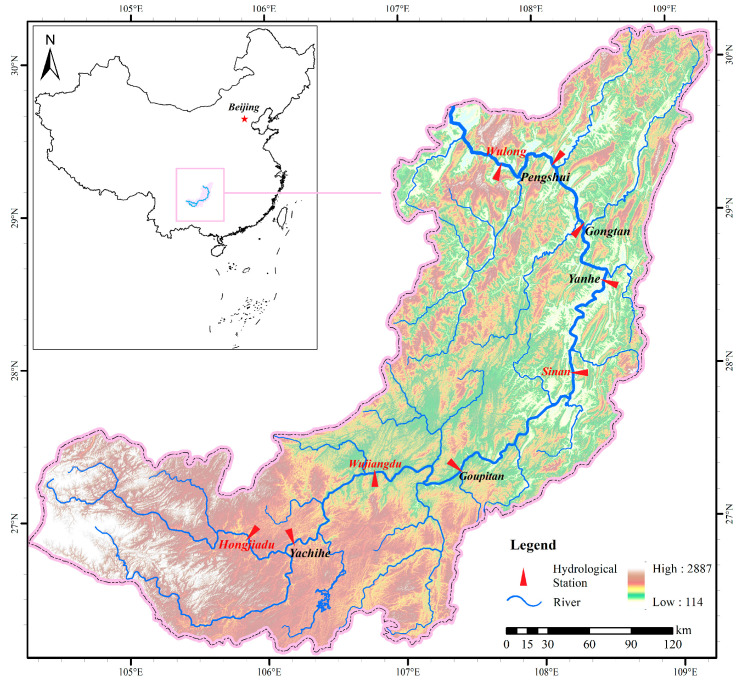
Map of the Wujiang River Basin.

The Wujiang River Basin has a natural drop of 2124 m and an average channel gradient of 0.205%, rich in hydropower resources. Since the 1970s, China carried out large-scale hydropower development in the Wujiang River Basin and planned a 12-level development scheme in the mainstream. Except for the Baima navigation and hydropower project at the most downstream area, all the planned hydraulic engineering projects are completed, and the impact on the ecological environment is gradually emerging. The geographical location and water system distribution of the Wujiang River Basin are shown in Figure 1.

In this study, the daily runoff data of critical hydrological stations in the Wujiang River Basin (Figure 1) from the past 64 years (1956~2019) were referenced from the *Hydrological Yearbook of the People’s Republic of China* [32]. The environmental flow was calculated using the collected data.

Considering the spatial variability of environmental flow, comprehensively evaluated from the aspects of hydraulic connection, representativeness, monitoring degree and adjustment degree, this study identified four critical cross sections located downstream of the corresponding reservoir for ecological protection along the mainstream of the Wujiang River, as shown in Table 1, the name of which is marked as red in Figure 1.

## 3. Methodology

**Figure 2:**
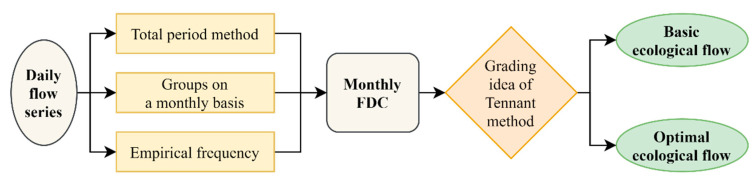
Technical route of the T-FDC method.

The T-FDC method proposed in this paper is characterized by drawing on the classification idea of the Tennant method to obtain multi-level environmental flows. Figure 2 presents the design framework for the proposed T-FDC method.

### 3.1. Monthly FDC

Generally, the T-FDC method prioritizes daily flow series and constructs FDCs month by month based on the total period method, provided that data are available to improve the time variability and accuracy of the method. Weekly and monthly flow series can also be used in case of poor data availability, making the method flexible.

To address the deficit of the calculated environmental flow, which is a single value within or between years. The specific guarantee rate has greater empirical and regional applicability from constructing curves on an annual scale [12]. This paper groups flow data on a monthly basis (e.g., “all the Mays” within a range of years) and constructs the FDC on a month-by-month basis [33] to enhance the representation of the temporal variability of the flow regime.

Depending on the different processing of runoff data, major approaches to constructing FDC include the total period approach, the calendar year approach, and the median annual approach [34]. The total period approach can comprehensively describe the basin’s historical runoff changes. The calendar year approach and the median annual approach have advantages in describing the interannual variation in runoff and reducing the impact of extreme flow events. However, they significantly shorten the daily average flow series length, resulting in increased uncertainty in the description of the monthly variation characteristics of runoff by the FDC [35].

The time scale of flow points for constructing the FDC is self-defined according to management requirements, which can be daily, weekly, or longer. However, the larger the time scale is, the easier it is to blur the details of flow changes, especially in small flow areas, such as sources and tributaries. The difference between FDCs constructed based on daily flow versus monthly flow may be as high as 35% [36].

There are many methods for calculating curves. Some classical ones include the technique based on the empirical frequency and the method based on information entropy theory proposed by V. P. Singh [37], and so on. These methods have their statistical basis, and the results are consistent. The FDC based on entropy theory can resist the influence of extreme values, but its estimation method is more complex. Therefore, considering the simplicity of the technique, this paper selects the empirical frequency to construct a monthly FDC:

(1) $$P_{i}=\frac{i}{j+1}$$

where *P_i_* is the empirical frequency of a measured daily flow in a specific month, *i* is the descending number of all daily flows of the month in all years, and *j* is the sample size (i.e., the number of days of the month in all years).

For the FDC of each month, different distributions, such as polynomial function, rational function, power function [38,39] and others, are used to fit the data and estimate corresponding parameters. The reliability and practicability of the fitting results are evaluated by the coefficient of determination (R^2^) to get the best estimates of the parameters.

### 3.2. Basic Environmental Flow

The basic environmental flow refers to the minimum value of discharge that needs to be maintained in a river to ensure basic conditions for existing ecosystems [40], according to appropriate criteria based on hydrological and environmental conditions.

The flow *Q*_90_ or *Q*_95_ at 90% or 95% of the period of record is often selected as the basic environmental flow in the FDC method [41,42]. However, the specific positioning of *Q*_90_ and *Q*_95_ in terms of river ecosystem function is ambiguous in existing studies, and their adaptability varies across rivers. Therefore, to improve the transferability of the basic environmental flow index, the T-FDC method selects the mean value of *Q*_90_ and *Q*_95_ as the basic environmental flow. For a specific month, the basic environmental flow is:

(2) $$E_{\min}=\frac{Q_{90}+Q_{95}}{2}$$

where *E*_min_ is the basic environmental flow, *Q*_90_ and *Q*_95_ are the flow at the 90% and 95% period of the record on the FDC of the corresponding month, respectively.

### 3.3. Optimal Environmental Flow

The optimal environmental flow refers to the runoff process of maintaining a sound ecological condition of the water body, which is the control index for developing and utilizing water resources.

Using predetermined percentages of average annual flow as environmental flow criteria (Table 2), the Tennant method divides the ecological status into ten levels based on arithmetic progression. It takes the 6th~10th level as the optimal ecological state. This idea of grading is a bright spot in all environmental flow calculation methods.

For the deficiency that the specific guarantee rate has a greater degree of empirical and regional applicability to the traditional FDC method, the grading idea of the Tennant method is borrowed and combined with the concept of guarantee rate of the FDC method. Taking the flow at 50% of the period of record on the FDC as the upper limit of the optimal environmental flow [16,43] divides the range from the basic environmental flow to the upper limit into ten levels, and 6th~10th levels are taken as the optimal ecological range, of which the 6th level is the lower limit, that is:

(3) $$\begin{array}{c} E_{opt}=\left[E_{opt.lower},E_{opt.upper}\right]\\ \left\{\begin{array}{l} E_{opt.upper}=Q_{50}\\ E_{opt.lower}=\frac{E_{opt.upper}-E_{\min}}{9}\times 5+E_{\min}=\frac{5}{9}Q_{50}+\frac{4}{9}E_{\min} \end{array}\right. \end{array}$$

where *E_opt_* is the optimal environmental flow range, *E_opt.lower_* and *E_opt.upper_* are the lower and upper limits of the range, respectively, *Q*_50_ is the flow at the 50% period of the record on the FDC of the corresponding month, and *E_min_* is the basic environmental flow defined by Equation (2).

### 3.4. Multi-Level Environmental Flow

As shown in Table 2, the arithmetic progression of the Tennant method is based on the percentage of the river flow in the natural multi-year average. To avoid the impact of extreme wet or dry events, modify the difference value between the basic environmental flow and the lower limit of optimal environmental flow to 10% of the monthly *Q*_50_. Then, the number of levels from basic environmental flow to the lower bound of the optimal range in a specific month of any particular section is:

(4) $$n=\langle \frac{E_{opt.lower}-E_{\min}}{0.1Q_{50}}\rangle +1=\langle \frac{5}{9}\times \frac{Q_{50}-E_{\min}}{0.1Q_{50}}\rangle +1$$

where *n* is the number of levels, 〈 〉 represents the rounding operation.

To facilitate the actual engineering operation, the number of environmental flow levels in each month of the same section should preferably be a fixed value. For taking one from different values, there can be options for taking the minimum, the maximum, the average, and the mode. In practical engineering, a smaller number of grades is more convenient for operation, so the maximum value is unsuitable. Whereas the minimum does not cater for the rest of the months in statistical significance, and the mode is a better indicator of the most demand, rather than the average. Therefore, take the mode of each month’s level number as the final environmental flow level number of the corresponding section. If the mode is not unique, then take the smallest integer greater than or equal to the average of these modes, that is:

(5) $$N=\left\lceil \bar{\mathrm{Mo}\left\{n\right\}}\right\rceil$$

where *N* is the final level number from the basic to lower bound of a specific section; {*n*} is the set of level numbers for each month of the section; and Mo and ⌈ ⌉ represent the mode and ceiling operations, respectively.

Ultimately, based on the grading idea of the Tennant method, the environmental flow at all levels from basic to lower bound in a particular month is:

(6) $$E_{m}=E_{\min}+\frac{5}{9}\times \frac{m-1}{N-1}\times \left(Q_{50}-E_{\min}\right)\left(m=1,2,\cdots ,N\right)$$

where *E_m_* is the *m*-th level environmental flow. *E*_1_ is the basic environmental flow, *E_N_* is the lower limit of the optimal environmental flow range, and *Q*_50_ is the upper limit of the range.

## 4. Results and Discussion

### 4.1. Calculation Results of Multi-Level Environmental Flow

**Figure 3:**
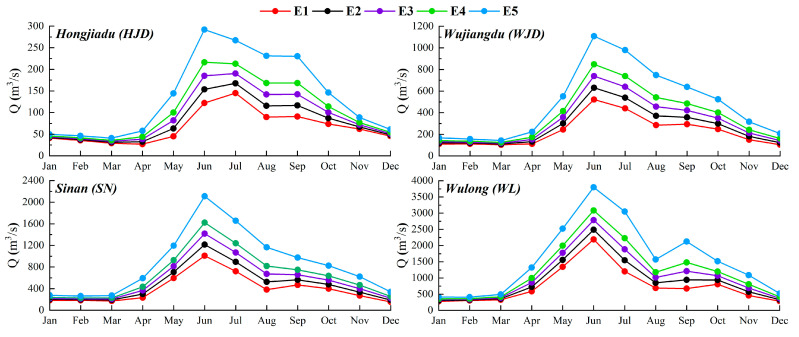
Multi-level environmental flow processes of Wujiang River.

Based on the historical daily runoff data, the monthly FDCs of each critical section were fitted, respectively, (regarding Appendix A, Appendix B, Appendix C and Appendix D for details). Then the environmental flow with different levels at separate sections was calculated and compared. More specifically, in terms of the calculated multi-level environmental flow, there are five environmental flow levels at each section (HJD, WJD, SN, and WL, four sections in total), respectively, *E*_1_~*E*_5_, of which are recorded in Table 3, and vividly shown in Figure 3. Where *E*_1_ is the basic environmental flow, *E*_2_ and *E*_3_ are the second and third levels of environmental flow representing, respectively, fair and good ecological status, and *E*_4_ and *E*_5_ are the lower and upper limit of the optimal environmental flow, respectively.

It turns out that the results of the multi-level environmental flow calculated through the T-FDC method proposed in this paper present pronounced monthly variability and level-by-level disparity. The variation shows a reasonable correlation with the wet and dry seasons of the Wujiang River Basin, which is abundant from May to September, and preliminarily proves the feasibility of the calculation method.

In addition, concerning the multi-level environmental flow of the Wujiang River calculated through the T-FDC method, there are five levels for each section, while there are seven levels for each region with the Tennant method applied. As a matter of fact, during actual management operation, the more environmental flow levels are taken into consideration, the more difficult it becomes for the actual reservoir operation and water resource allocation. Therefore, the T-FDC method gained an edge over the other in this case.

**Figure 4:**
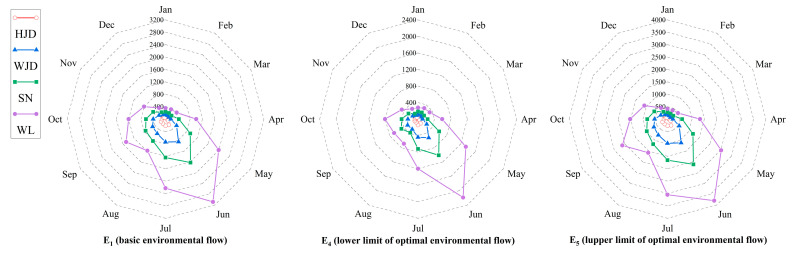
Environmental flow processes of each section.

Figure 4 shows the values of basic environmental flow and the upper and lower limits of optimal environmental flow at different sections. In addition to the apparent monthly variability shown in the figure, the values of environmental flow calculated by the T-FDC method from upstream to downstream of the Wujiang River vary considerably, which exhibits good spatial variability. Furthermore, the similar pattern (enclosing shape) of the environmental flow radar plots for different levels at each section obtained through the T-FDC method reflects its stability.

### 4.2. Comparison in Terms of Basic Environmental Flow

To further assess the validity of the T-FDC method in the Wujiang River Basin, some typical hydrological methods were selected for comparison. Among the existing methods, only the environmental flow obtained through the Tennant method has multiple levels, while the rest merely focus on the basic environmental flow. In addition, with the common methods implemented, including the traditional Tennant method, basic environmental flows of constant value within the year are obtained, lacking monthly variation. Therefore, for the basic environmental flow calculation, the improved monthly Tennant method [25] and the dynamic calculation method (DC) [44], which is widely used in China and recommended by the Chinese National Standard (SL/T 712-2021), were chosen for comparison with the T-FDC proposed in this paper. The improved monthly Tennant method was selected for comparison for the optimal environmental flow calculation.

**Figure 5:**
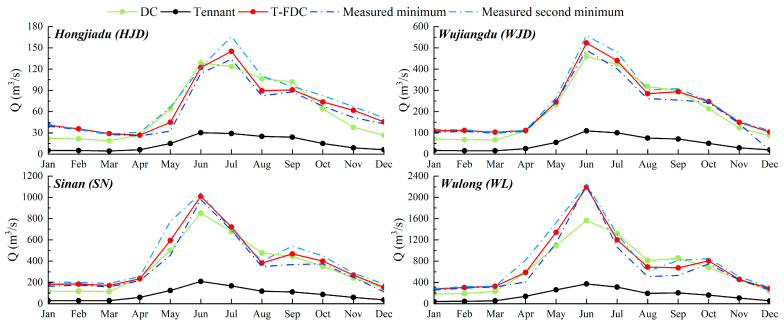
Basic environmental flow calculated by various methods and measured small flow.

Together with the historical minimum and second minimum monthly average flow process (MMAF and MMAF-II) in the measured runoff of the Wujiang River, the obtained results of the basic environmental flow calculation with the T-FDC method, the monthly Tennant method, and the DC method, respectively, applied were plotted as in Figure 5.

The basic environmental flow process by the T-FDC method is relatively stable, ranging between the historical MMAF and MMAF-II. In contrast, the basic environmental flow by the Tennant method turns out to be much lower than the natural MMAF, which is very unfavourable to the maintenance of river ecological health. This variability from low natural flows is due to the vulnerability of the Tennant method to the extremes of the natural hydrological situation, showing its poor spatial portability, which is not suitable for the Wu River.

Compared with the Tennant method, the environmental flow process calculated by the DC method shows better monthly variability, with values closer to the natural small flow process. Notwithstanding, compared with the T-FDC method, the values obtained by the DC method are generally smaller. Additionally, the values through such methods are significantly lower than the natural MMAF during the dry months of the year and higher than those by the T-FDC method and the natural MMAF-II at the end of the flood season. For the actual reservoir operation management, this is not conducive to ecological protection during the dry season and the realization of power generation benefits at the end of the flood season. Therefore, in comparison, the basic environmental flow process by the T-FDC method is more feasible and reasonable.

### 4.3. Comparison in Terms of Optimal Environmental Flow

**Figure 6:**
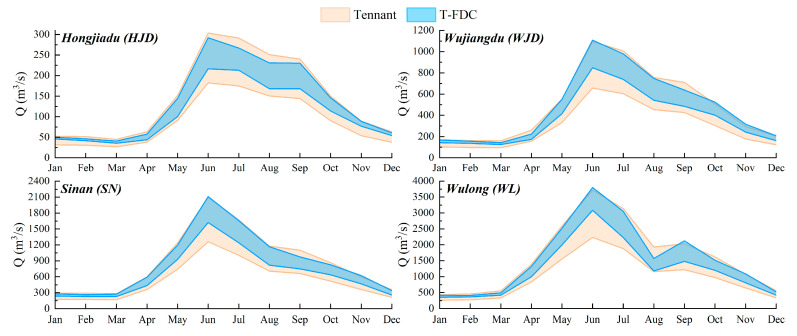
Optimal environmental flow calculated by two methods.

The optimal environmental flow results by the T-FDC method and the improved Tennant method are shown in Figure 6.

Overall, in the Wujiang River Basin, the T-FDC method’s optimal flow range covers that of the Tennant method. In addition, the ranges of optimal flow by the two methods are similar in the dry season, but the lower limit by the Tennant method is much lower than that by the T-FDC method in flood season. This is because the water volume changes drastically in flood season, which is prone to inducing extreme flow events, causing the mean value to deviate from the overall distribution and thus leading to a wide range of variations in the optimal environmental flow through applying the Tennant method. Thus, the T-FDC method proves more scientific and reasonable than the Tennant method for the ecosystem management in the Wujiang River.

## 5. Conclusions

This paper studied the development of environmental flow management objectives in the Wujiang River, with the most fundamental problems of the traditional hydrological methods pertinent to the subject analyzed. In an effort to address the problems thereof, a T-FDC method was proposed by combining the FDC method’s guarantee rate idea with the Tennant method’s grading idea. The results show its effectiveness and feasibility:

1. The T-FDC method is well applicable in the Wujiang River, showing monthly variability and spatial transferability, excluding the influence of extreme wet or dry events. Furthermore, the basic environmental flow process obtained through this method is stable, ranging between the historical minimum and the second minimum monthly average flow. In contrast, the threshold width of the optimal environmental flow is more reasonable than the Tennant method.
2. Only the measured runoff data, which can be on any time scale, is needed as the input required for the T-FDC method, which helps the handling of shortage for daily runoff processes and eliminates errors caused by interpolation, effectively improving the method’s adaptability.
3. Regarding practical engineering management, the proposed T-FDC method improved operability by reducing the number of environmental flow levels taken into consideration.

Several improvements can be incorporated into our proposed method. First, although the T-FDC method achieved successful application in the Wujiang River Basin, it is still necessary to further explore the applicability to rivers with different hydraulic conditions other than the Wujiang River and to evaluate the robustness, reproducibility, and spatial portability of the method. In addition, the T-FDC method proposes not to distinguish the interannual variation in wet and dry from the point of view of the operational simplicity of practical management. It can potentially result in too high a standard in dry years, which is challenging to operate in actuality and can moderately enhance the method’s adaptability to synchronous wet/dry events.

## Figures and Tables

**Table 1:** Critical sections for ecological protection on the Wujiang River.

Cross Section Name	Section Type
Hongjiadu (HJD)	Upstream/midstream boundary section
Wujiangdu (WJD)	Controlled hydraulic engineering section
Sinan (SN)	Midstream/downstream boundary section
Wulong (WL)	Basin outlet section

**Table 2:** The ecological status grading system of the Tennant method [18].

Narrative Description of Flows	Recommended Base Flow Regimens of the Average Flow (%)	
	General Water Use Period	Fish Spawning Period
Optimum range	(60, 100]	(60–100]
Outstanding	[40, 60]	60
Excellent	[30, 40)	[50, 60)
Good	[20, 30)	[40, 50)
Fair or degrading	(10, 20)	[30, 40)
Poor or minimum	10	[10, 30)
Severe degradation	(0, 10)	(0, 10)

**Table 3:** Environmental flows of Wujiang River calculated by the T-FDC method.

		January	February	March	April	May	June	July	August	September	October	November	December
Hongjiadu	*E*_1_	40.82	35.68	29.03	26.85	44.92	122.15	145.02	89.53	90.78	73.68	61.88	45.56
	*E*_2_	42.49	37.61	31.20	32.57	63.36	153.59	167.63	115.73	116.59	87.10	66.84	48.42
	*E*_3_	44.16	39.54	33.38	38.30	81.79	185.02	190.24	141.94	142.40	100.51	71.81	51.27
	*E*_4_	45.83	41.47	35.55	44.02	100.23	216.46	212.85	168.15	168.21	113.93	76.77	54.13
	*E*_5_	49.84	46.11	40.77	57.76	144.48	291.91	267.12	231.05	230.15	146.13	88.69	60.99
Wujiangdu	*E*_1_	109.79	111.56	103.30	111.31	244.82	522.44	439.91	284.56	293.53	247.58	149.98	104.22
	*E*_2_	120.52	119.90	110.51	132.04	301.56	630.82	539.70	370.23	357.28	298.74	180.73	123.18
	*E*_3_	131.25	128.25	117.73	152.77	358.31	739.21	639.49	455.90	421.04	349.89	211.49	142.15
	*E*_4_	141.98	136.60	124.94	173.50	415.05	847.59	739.28	541.57	484.79	401.05	242.24	161.11
	*E*_5_	167.74	156.63	142.26	223.25	551.24	1107.71	978.78	747.18	637.81	523.82	316.05	206.63
Sinan	*E*_1_	183.74	184.69	171.06	234.04	594.67	1010.28	721.70	381.39	468.37	400.17	269.10	153.63
	*E*_2_	200.93	198.97	189.69	300.52	705.70	1213.71	894.73	526.61	562.10	478.82	334.40	187.87
	*E*_3_	218.11	213.26	208.33	367.00	816.73	1417.14	1067.77	671.83	655.83	557.48	399.70	222.12
	*E*_4_	235.30	227.55	226.97	433.48	927.76	1620.57	1240.81	817.05	749.56	636.14	464.99	256.36
	*E*_5_	276.55	261.85	271.70	593.03	1194.24	2108.80	1656.11	1165.57	974.51	824.92	621.71	338.55
Wulong	*E*_1_	277.43	304.44	327.20	588.73	1340.95	2190.71	1201.19	689.37	673.46	805.17	456.66	285.55
	*E*_2_	302.84	323.65	357.60	724.55	1559.16	2488.50	1543.55	852.14	942.09	936.91	573.25	329.41
	*E*_3_	328.26	342.86	388.00	860.37	1777.36	2786.30	1885.92	1014.91	1210.73	1068.64	689.83	373.26
	*E*_4_	353.67	362.08	418.41	996.20	1995.57	3084.09	2228.29	1177.68	1479.36	1200.38	806.42	417.12
	*E*_5_	414.67	408.19	491.38	1322.17	2519.26	3798.80	3049.97	1568.34	2124.09	1516.55	1086.22	522.38

**Figure A1:**
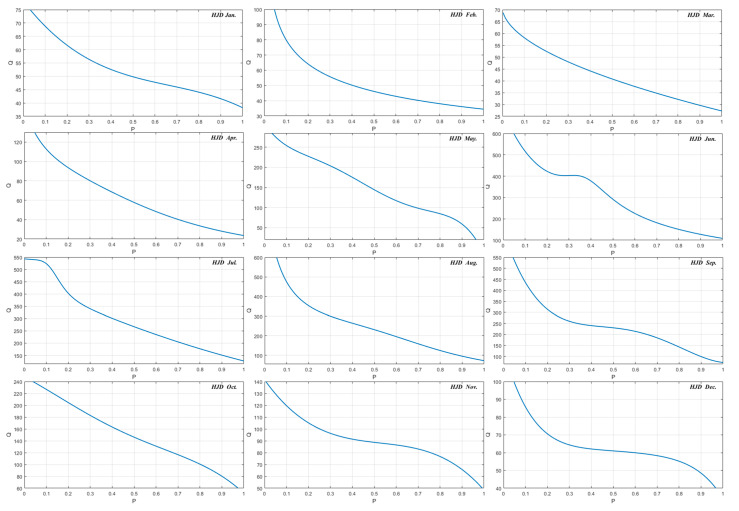
Monthly fitted FDC of Hongjiadu section.

**Figure A2:**
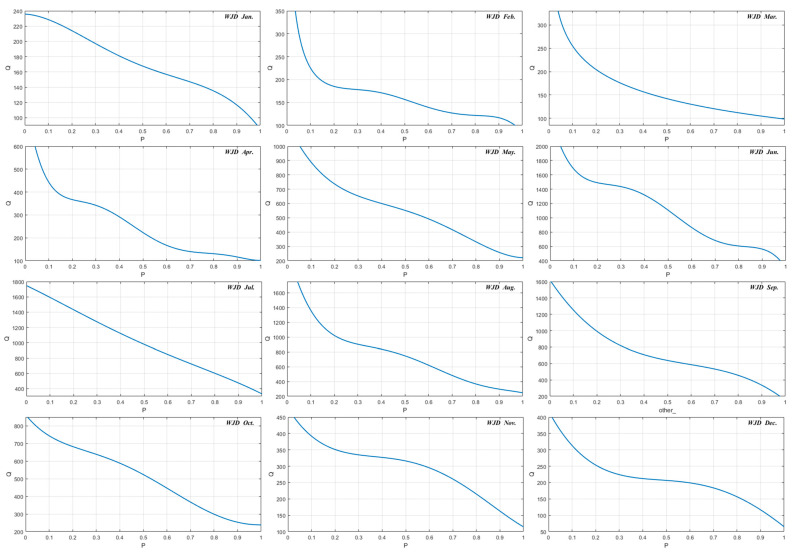
Monthly fitted FDC of Wujiangdu section.

**Figure A3:**
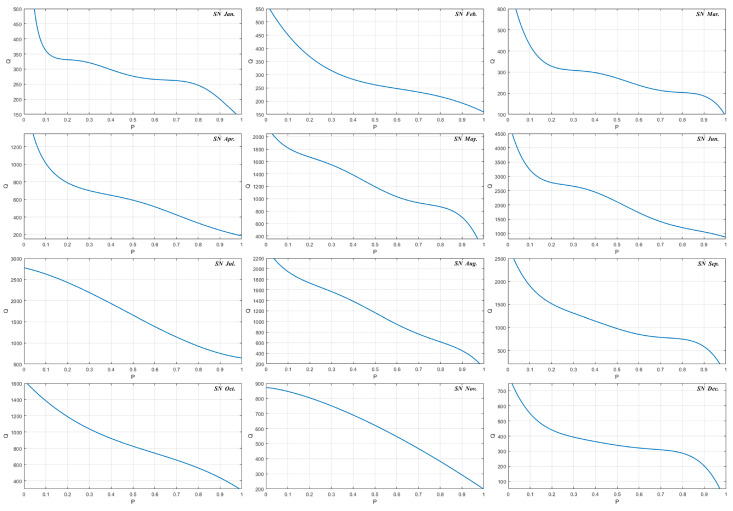
Monthly fitted FDC of Sinan section.

**Figure A4:**
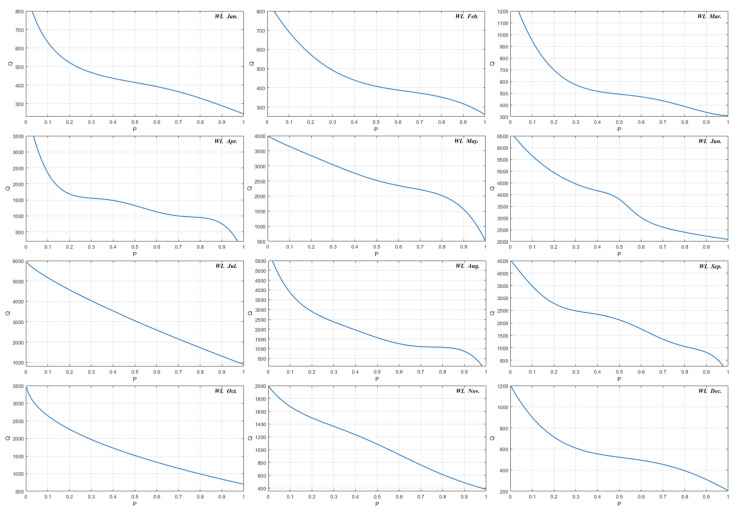
Monthly fitted FDC of Wulong section.

## Data Availability

The data that support the findings of this study are available from the corresponding author upon reasonable request.

## Appendix A. Fitted FDC of Hongjiadu

**Table A1:** Fit type and equation form of Hongjiadu section.

Month	Fit Type	Degree		Equation Form
		Numerator	Denominator	
January	Polynomial	3		$f\left(x\right)=a_{1}x^{3}+a_{2}x^{2}+a_{3}x+a_{4}$
February	Power	-		$f\left(x\right)=a_{1}x^{a_{2}}+a_{3}$
March	Power	-		$f\left(x\right)=a_{1}x^{a_{2}}+a_{3}$
April	Rational	1	3	$f\left(x\right)=\frac{a_{1}x+a_{2}}{x^{3}+a_{3}x^{2}+a_{4}x+a_{5}}$
May	Polynomial	5		$f\left(x\right)=a_{1}x^{5}+a_{2}x^{4}+a_{3}x^{3}+a_{4}x^{2}+a_{5}x+a_{{}_{6}}$
June	Rational	3	3	$f\left(x\right)=\frac{a_{1}x^{3}+a_{2}x^{2}+a_{3}x+a_{4}}{x^{3}+a_{5}x^{2}+a_{6}x+a_{7}}$
July	Rational	4	2	$f\left(x\right)=\frac{a_{1}x^{4}+a_{2}x^{3}+a_{3}x^{2}+a_{4}x+a_{5}}{x^{2}+a_{6}x+a_{7}}$
August	Rational	0	3	$f\left(x\right)=\frac{a_{1}}{x^{3}+a_{2}x^{2}+a_{3}x+a_{4}}$
September	Polynomial	4		$f\left(x\right)=a_{1}x^{4}+a_{2}x^{3}+a_{3}x^{2}+a_{4}x+a_{5}$
October	Polynomial	4		$f\left(x\right)=a_{1}x^{4}+a_{2}x^{3}+a_{3}x^{2}+a_{4}x+a_{5}$
November	Polynomial	3		$f\left(x\right)=a_{1}x^{3}+a_{2}x^{2}+a_{3}x+a_{4}$
December	Polynomial	5		$f\left(x\right)=a_{1}x^{5}+a_{2}x^{4}+a_{3}x^{3}+a_{4}x^{2}+a_{5}x+a_{{}_{6}}$

**Table A2:** Specific parameters of Hongjiadu section.

Month	*a*_1_	*a*_2_	*a*_3_	*a*_4_	*a*_5_	*a*_6_	*a*_7_	R^2^
January	−66.96	134	−107.1	78.25	-	-	-	0.981
February	82.21	−0.1915	−47.76	-	-	-	-	0.988
March	−43.62	0.5355	70.87	-	-	-	-	0.991
April	19.39	9.285	−0.2621	0.42	0.05906			0.982
May	−4990	11,720	−9801	3562	−811.8	308.4	-	0.993
June	−83.85	269	−154.4	30.21	−0.4225	−0.05657	0.04252	0.995
July	112.7	−488.5	599	−137.6	13.11	−0.2484	0.02413	0.992
August	39.36	−1.019	0.5199	0.04017	-	-	-	0.974
September	3608	−9135	7943	−3007	664.3	-	-	0.973
October	−360.1	575.9	−219.9	−192.2	247.7	-	-	0.993
November	−285.8	453.8	−261.1	141.5	-	-	-	0.976
December	−1.284	1.546	−2.555	−0.1121	−2.637	61	-	0.980

## Appendix B. Fitted FDC of Wujiangdu

**Table A3:** Fit type and equation form of Wujiangdu section.

Month	Fit Type	Degree		Equation Form
		Numerator	Denominator	
January	Polynomial	4		$f\left(x\right)=a_{1}x^{4}+a_{2}x^{3}+a_{3}x^{2}+a_{4}x+a_{5}$
February	Rational	1	5	$f\left(x\right)=\frac{a_{1}x}{x^{5}+a_{2}x^{4}+a_{3}x^{3}+a_{4}x^{2}+a_{5}x+a_{6}}$
March	Power	-	-	$f\left(x\right)=a_{1}x^{a_{2}}+a_{3}$
April	Polynomial	6		$f\left(x\right)=a_{1}x^{6}+a_{2}x^{5}+a_{3}x^{4}+a_{4}x^{3}+a_{5}x^{2}+a_{{}_{6}}x+a_{7}$
May	Polynomial	4		$f\left(x\right)=a_{1}x^{4}+a_{2}x^{3}+a_{3}x^{2}+a_{4}x+a_{5}$
June	Polynomial	5		$f\left(x\right)=a_{1}x^{5}+a_{2}x^{4}+a_{3}x^{3}+a_{4}x^{2}+a_{5}x+a_{{}_{6}}$
July	Polynomial	4		$f\left(x\right)=a_{1}x^{4}+a_{2}x^{3}+a_{3}x^{2}+a_{4}x+a_{5}$
August	Polynomial	5		$f\left(x\right)=a_{1}x^{5}+a_{2}x^{4}+a_{3}x^{3}+a_{4}x^{2}+a_{5}x+a_{{}_{6}}$
September	Polynomial	4		$f\left(x\right)=a_{1}x^{4}+a_{2}x^{3}+a_{3}x^{2}+a_{4}x+a_{5}$
October	Polynomial	5		$f\left(x\right)=a_{1}x^{5}+a_{2}x^{4}+a_{3}x^{3}+a_{4}x^{2}+a_{5}x+a_{{}_{6}}$
November	Polynomial	4		$f\left(x\right)=a_{1}x^{4}+a_{2}x^{3}+a_{3}x^{2}+a_{4}x+a_{5}$
December	Polynomial	4		$f\left(x\right)=a_{1}x^{4}+a_{2}x^{3}+a_{3}x^{2}+a_{4}x+a_{5}$

**Table A4:** Specific parameters of Wujiangdu section.

Month	*a*_1_	*a*_2_	*a*_3_	*a*_4_	*a*_5_	*a*_6_	*a*_7_	R^2^
January	−667.7	1212	−679.2	−16.14	235.8	-	-	0.996
February	−4.157	5.794	−2.738	2.771	−1.279	0.2573	0.00839	0.997
March	678.8	−0.09016	−580.3	-	-	-	-	0.985
April	35,450	−126,600	175,600	−118,600	40,140	−6766	820.6	0.997
May	4667	−11,000	8890	−3498	1161	-	-	0.984
June	−58,890	158,000	−152,900	64,400	−12,720	2448	-	0.983
July	−1131	2057	−865.1	−1479	1748	-	-	0.991
August	−22,210	71,200	−84,760	46,120	−12,310	2209	-	0.986
September	203.2	−4012	6658	−4322	1623	-	-	0.950
October	−3179	10,900	−12,690	6022	−1683	863.9	-	0.990
November	1796	−4439	3435	−1155	477.3	-	-	0.987
December	986.5	−3174	3176	−1342	418.7	-	-	0.978

## Appendix C. Fitted FDC of Sinan

**Table A5:** Fit type and equation form of Sinan section.

Month	Fit Type	Degree		Equation Form
		Numerator	Denominator	
January	Rational	2	5	$f\left(x\right)={\frac{a_{1}x^{2}+a_{2}x+a_{3}}{x^{5}+a_{4}x^{4}+a_{5}x^{3}+a_{6}x^{2}+a_{7}x+a}}_{8}$
February	Polynomial	4		$f\left(x\right)=a_{1}x^{4}+a_{2}x^{3}+a_{3}x^{2}+a_{4}x+a_{5}$
March	Polynomial	5		$f\left(x\right)=a_{1}x^{5}+a_{2}x^{4}+a_{3}x^{3}+a_{4}x^{2}+a_{5}x+a_{{}_{6}}$
April	Rational	0	3	$f\left(x\right)=\frac{a_{1}}{x^{3}+a_{2}x^{2}+a_{3}x+a_{4}}$
May	Polynomial	5		$f\left(x\right)=a_{1}x^{5}+a_{2}x^{4}+a_{3}x^{3}+a_{4}x^{2}+a_{5}x+a_{{}_{6}}$
June	Polynomial	6		$f\left(x\right)=a_{1}x^{6}+a_{2}x^{5}+a_{3}x^{4}+a_{4}x^{3}+a_{5}x^{2}+a_{{}_{6}}x+a_{7}$
July	Polynomial	3		$f\left(x\right)=a_{1}x^{3}+a_{2}x^{2}+a_{3}x+a_{4}$
August	Polynomial	5		$f\left(x\right)=a_{1}x^{5}+a_{2}x^{4}+a_{3}x^{3}+a_{4}x^{2}+a_{5}x+a_{{}_{6}}$
September	Polynomial	5		$f\left(x\right)=a_{1}x^{5}+a_{2}x^{4}+a_{3}x^{3}+a_{4}x^{2}+a_{5}x+a_{{}_{6}}$
October	Polynomial	3		$f\left(x\right)=a_{1}x^{3}+a_{2}x^{2}+a_{3}x+a_{4}$
November	Power	-		$f\left(x\right)=a_{1}x^{a_{2}}+a_{3}$
December	Polynomial	5		$f\left(x\right)=a_{1}x^{5}+a_{2}x^{4}+a_{3}x^{3}+a_{4}x^{2}+a_{5}x+a_{{}_{6}}$

**Table A6:** Specific parameters of Sinan section.

Month	*a*_1_	*a*_2_	*a*_3_	*a*_4_	*a*_5_	*a*_6_	*a*_7_	*a*_8_	R^2^
January	−0.2816	8.041	1.938	−2.172	1.72	−0.5942	0.1184	0.00016	0.997
February	424.3	−1850	2446	−1434	572.1	-	-	-	0.964
March	−16,530	45,480	−46,880	22,300	−5048	752.7	-	-	0.973
April	82.98	−1.086	0.4871	0.04298	-	-	-	-	0.992
May	−40,600	95,210	−79,690	28,900	−5878	2189	-	-	0.992
June	82,660	−337,800	535,300	−411,700	157,400	−29,970	5020		0.988
July	2342	−3297	−1178	2777	-	-	-	-	0.986
August	−27,420	70,330	−65,500	26,930	−6627	2396	-	-	0.982
September	−49,260	122,600	−115,100	52,050	−13,160	2802	-	-	0.994
October	−1890	3371	−2837	1637	-	-	-	-	0.987
November	−673.4	1.43	871.6	-	-	-	-	-	0.996
December	−13,120	32,160	−30,790	14,670	−3779	808.4	-	-	0.989

## Appendix D. Fitted FDC of Wulong

**Table A7:** Fit type and equation form of Wulong section.

Month	Fit Type	Degree		Equation Form
		Numerator	Denominator	
January	Rational	0	3	$f\left(x\right)=\frac{a_{1}}{x^{3}+a_{2}x^{2}+a_{3}x+a_{4}}$
February	Polynomial	3		$f\left(x\right)=a_{1}x^{3}+a_{2}x^{2}+a_{3}x+a_{4}$
March	Polynomial	4		$f\left(x\right)=a_{1}x^{4}+a_{2}x^{3}+a_{3}x^{2}+a_{4}x+a_{5}$
April	Polynomial	5		$f\left(x\right)=a_{1}x^{5}+a_{2}x^{4}+a_{3}x^{3}+a_{4}x^{2}+a_{5}x+a_{{}_{6}}$
May	Polynomial	5		$f\left(x\right)=a_{1}x^{5}+a_{2}x^{4}+a_{3}x^{3}+a_{4}x^{2}+a_{5}x+a_{{}_{6}}$
June	Rational	2	3	$f\left(x\right)={\frac{a_{1}x^{2}+a_{2}x+a_{3}}{a_{4}x^{3}+a_{5}x^{2}+a_{6}x+a}}_{7}$
July	Power	-		$f\left(x\right)=a_{1}x^{a_{2}}+a_{3}$
August	Polynomial	5		$f\left(x\right)=a_{1}x^{5}+a_{2}x^{4}+a_{3}x^{3}+a_{4}x^{2}+a_{5}x+a_{{}_{6}}$
September	Polynomial	6		$f\left(x\right)=a_{1}x^{6}+a_{2}x^{5}+a_{3}x^{4}+a_{4}x^{3}+a_{5}x^{2}+a_{{}_{6}}x+a_{7}$
October	Power	-		$f\left(x\right)=a_{1}x^{a_{2}}+a_{3}$
November	Rational	0	3	$f\left(x\right)=\frac{a_{1}}{x^{3}+a_{2}x^{2}+a_{3}x+a_{4}}$
December	Polynomial	4		$f\left(x\right)=a_{1}x^{4}+a_{2}x^{3}+a_{3}x^{2}+a_{4}x+a_{5}$

**Table A8:** Specific parameters of Wulong section.

Month	*a*_1_	*a*_2_	*a*_3_	*a*_4_	*a*_5_	*a*_6_	*a*_7_	R^2^
January	142.2	−1.399	0.8248	0.1555	-	-	-	0.978
February	−1413	2721	−1904	856.6	-	-	-	0.987
March	5427	−14,370	13,500	−5629	1390	-	-	0.984
April	−118,500	317,500	−320,100	150,100	−33,660	4504	-	0.992
May	−24,740	44,050	−25,890	7044	−3954	3992	-	0.997
June	3239	−3426	960.5	−0.5323	−0.2408	0.1435	-	0.995
July	−5084	0.7955	5980	-	-	-	-	0.993
August	−107,600	274,200	−262,600	121,300	−31,520	6044	-	0.988
September	−198,300	547,900	−541,700	214,700	−16,700	−10,460	4526	0.997
October	−2962	0.4657	3661	-	-	-	-	0.986
November	312.7	−0.6969	0.3614	0.1564	-	-	-	0.993
December	2364	−7650	8070	−3784	1206	-	-	0.976
